# Response: Commentary: Quantile treatment effect of zinc lozenges on common cold duration: a novel approach to analyze the effect of treatment on illness duration

**DOI:** 10.3389/fphar.2024.1335784

**Published:** 2024-04-09

**Authors:** Harri Hemilä, Elizabeth Chalker, Janne Tukiainen

**Affiliations:** ^1^ Department of Public Health, University of Helsinki, Helsinki, Finland; ^2^ National Centre for Epidemiology and Population Health, Australian National University, Canberra, Australia; ^3^ Department of Economics, University of Turku, Turku, Finland

**Keywords:** data interpretation, outcome assessment (healthcare), quantile regression (QR), quantile treatment effect, randomized controlled trial, the common cold, treatment outcome, zinc lozenges

## Introduction

Quantile regression is a standard statistical method which is widely used in econometrics (Binder and Coad, 2011; Chen et al., 2014; Koenker, 2017). Its use in clinical medicine has been encouraged (Beyerlein, 2014; Hong et al., 2019; Staffa et al., 2019). The quantile treatment effect (QTE) can be estimated with quantile regression and QTE can be useful in the analysis of randomized controlled trials since it enables investigation of treatment effects over the whole distribution of a continuous outcome, and not just the average treatment effect (ATE) (Schiele and Schmitz, 2016; Ohrnberger et al., 2020; Hemilä et al., 2021; Callaway, 2022; Hemilä and Pirinen, 2023; Pirinen and Hemilä, 2023).

In this journal, we used the QTE to analyze the effect of zinc lozenges on common cold duration and encouraged its use in the analysis of randomized controlled trials (Hemilä et al., 2022). In clinical medicine, there is usually greater interest in the effect of a treatment on longer illness duration than shorter. We selected zinc lozenges and the common cold as the example, given in this context illness duration is a particularly relevant health outcome.

## Commentary on our paper

Jonas Moss (Moss, 2023) criticizes our paper with the first argument being that:

“*we would have liked to know the conditional average treatment effect* [his Eq. (1)]*. But this and similar conditional quantities, such as conditional medians, are impossible to estimate from randomized clinical trials alone, as they depend on the joint distribution of T*_*placebo*_ *and T*_*treatment*_.”

Our paper was concerned with the quantile treatment effect (QTE), that is, Q(Y_i_(1))—Q(Y_i_(0)), where Y refers to the potential outcomes. Identification of the QTE does not require knowledge or assumptions regarding the joint distribution, but only being able to infer the marginal distributions. In the context of randomized controlled trials, the QTE is identified. This is because under randomization of treatment, the partially unobserved distribution of treated potential outcomes is the same as the fully observed distribution of realized outcomes for the treated group; and likewise, the partially unobserved distribution of untreated potential outcomes is the same as the fully observed distribution of realized outcomes for the untreated group (Callaway, 2022).

However, the QTE is not the same as quantiles of the treatment effect Q(Y_i_(1)—Y_i_ (0)). Perhaps Moss was referring to that quantity as identifying it does require potentially strong assumptions about the joint distribution such as rank invariance (individuals maintain their expected ranks in the potential outcomes distributions). However, although the rank invariance assumption enriches the interpretation of quantile regression results, the QTE also has a meaningful interpretation without it. Specifically, the QTEs can be interpreted as comparing the same quantile in the treated and untreated distributions, rather than individuals occupying different positions within the potential outcome distributions (Firpo, 2007; Melly et al., 2017). Concerning the zinc lozenges, this would mean comparing, for example, the 95th percentile cold duration among patients with the corresponding 95th percentile in the contrafactual scenario, where the same patients may or may not occupy the 95th percentile in the two potential outcomes distributions (Borgen et al., 2023).

Moreover, Jonas Moss (Moss, 2023) criticized our statement (Hemilä et al., 2022):

*“… the [QTE] analysis* [of the Mossad et al. (1996)] *indicates that 15- to 17-day colds were shortened by 8 days, and 2-day colds by just 1 day, for the group taking zinc lozenges.”*

Moss argued that:

“This conclusion is too strong and potentially misleading, as the quantile treatment effect only indicates anything of the sort when quite stringent assumptions on the joint distribution of (T_treatment_, T_placebo_) are met.

… consider two patients with exactly the same cold duration, one who is 58 and male and one who is 17 and female. If the relationship between placebo outcome and treatment outcome is deterministic, both patients must have exactly the same cold duration when treated with zinc lozenge. This assumption is virtually guaranteed to be false.”

In QTE analysis, the observations in the control and treatment groups are ordered from the smallest to the largest, and on each quantile level of the control group the difference between the observations at that same quantile level gives the measure of effect as the QTE (Callaway, 2022; Hemilä et al., 2022).

To be precise, the QTE function is a measure of the difference between the two distributions of observations. The QTE at a specified quantile level does not indicate that each person with the same control-stage outcome level would have the same treatment effect when being treated. In this respect we agree with Moss: the above sentence is not accurate. The QTE does not equal the individual-level treatment effect (TE).

## Relationship between the QTE and the individual-level treatment effects

Although the QTE does not equal the individual-level TE, it is useful to consider their relationship. In the counterfactual context (potential outcomes context) (Rubin, 1974), let us assume that a person in the control stage has outcome X, while the same person in the treatment stage has outcome X*. We can write the variance of the individual-level TE (X* − X) in terms of correlation r = cor(X,X*), and variances v = var(X) of the untreated and v* = var(X*) of the treated stages, as follows:

$$\begin{array}{c} \text{var}\left(X*-X\right)=v+v*\;-2\text{ cov}\left(X,X*\right)\\ =v+v*-2\;r\;\sqrt{v}\cdot \sqrt{v*}. \end{array}$$

The minimum variance in the individual-level TE is reached when r = +1. It means that there is a constant perfect linear relationship between the treatment and control outcomes for all individuals. It is unlikely that any treatment behaves consistently in such a way, yet this gives the lower limit for the mathematically possible variance in the individual-level TEs. If we assume that treatment preserves the rank, this leads to the maximum r for the observed data. Under this assumption, the QTE is the minimum-variance approximation of the individual-level TE distribution for a data set.

The maximum variance in the individual-level TE is reached when r = −1. A consistent reversal of order is unlikely in real life, yet this gives the upper limit for the mathematically possible variance.

Empirical data gives the maximum and minimum possible values for r for a specific study. In the Mossad trial with zinc lozenges, r = +0.97 when assuming that the order is preserved, and r = −0.93 when assuming that the order is reversed.

**FIGURE 1:**
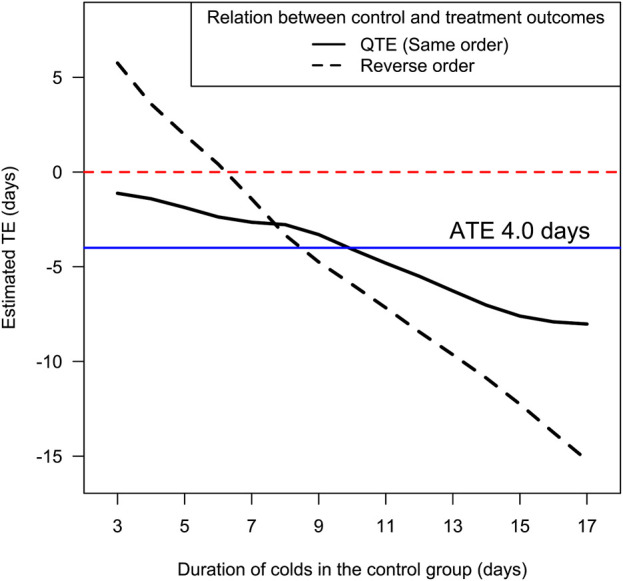
Estimated treatment effect of zinc lozenges in the Mossad trial by different assumptions of the order between the outcomes in the placebo and zinc lozenge groups. The two curves are constructed by assuming that the order of the zinc and placebo groups is fully preserved (QTE) or fully reversed. For a 15-day cold in the control group, the minimum possible expected average for the individual-level decrease in duration is 7.6 days and the maximum is 12.2 days. The curves were drawn with the BQTE program, which back-transforms the ordinary QTE estimates to the measurement units, in this case to the day scale (Hemilä and Pirinen, 2023; Pirinen and Hemilä, 2023). The Mossad data is available (Hemilä et al., 2022; Pirinen and Hemilä, 2023).

Figure 1 shows two curves assuming a different order between the outcomes in the control and treatment groups in the Mossad trial. The QTE curve retains the order and has the smallest deviation from the ATE of 4.0 days, whereas the curve of the reversed order has the greatest deviation from the ATE.

The QTE is a conservative approximation of the individual-level TE. Since the QTE corresponds to the minimum possible variance, it cannot be biased towards exaggerated variance in the individual-level TE. In real life, the order is not fully preserved and therefore the expected real individual-level TEs are on average further from the ATE than the QTE estimate. Thus, the real individual-level TEs have greater variance than that indicated by the QTE.

Figure 1 indicates that for a cold duration of 15 days in the control group, the expected average decrease in duration cannot be less than 7.6 days. Thus, to revise the sentence that Moss had concerns with, a more accurate statement is that 15-day colds were shortened on average by at least 7.6 days in the Mossad trial.

Separately, we have shown that the upper tail back-transformed quantile treatment effect (UTBQTE) gives an upper bound for the ATE of a selected upper tail region (Hemilä and Pirinen, 2023; Pirinen and Hemilä, 2023). This approach does not make any assumptions about whether the treatment preserves the ranking. Based on the UTBQTE analysis (Hemilä and Pirinen, 2023), another revision of the sentence that Moss criticized is that the ATE on colds lasting 15 days and longer is at least 5.7 days (based on 95% CI 5.7–9.8 days) in the Mossad trial.

## Conclusion

Although Moss is correct in noting that one of our sentences was potentially misleading, the inaccuracy has no material importance in the interpretation of the QTE curves in our paper (Hemilä et al., 2022). The QTE is identified and has a meaningful interpretation without extra assumptions. Moreover, the QTE gives the minimum possible deviation of individual-level TEs from the ATE. Thus, it is a conservative approximation of the variation around the ATE effect. The QTE analysis shows that a single ATE estimate poorly captures the effects of effective treatments (Hemilä et al., 2022). The QTE is a useful approach to analyze duration data of randomized trials.
